# Chemically Crosslinked Alginate Hydrogel with Polyaziridine: Effects on Physicochemical Properties and Promising Applications

**DOI:** 10.1002/cplu.202400649

**Published:** 2024-12-18

**Authors:** Chaehoon Lee, Giacomo Fiocco, Barbara Vigani, Teresa Recca, Chiara Milanese, Chiara Delledonne, Maurizio Licchelli, Silvia Rossi, Yongjae Chung, Francesca Volpi, Maduka L. Weththimuni, Marco Malagodi

**Affiliations:** ^1^ Department of Chemistry University of Pavia Via Taramelli 12 27100 Pavia Italy; ^2^ Arvedi Laboratory of Non-invasive Diagnostics University of Pavia via Bell'Aspa 3 26100 Cremona Italy; ^3^ Department of Musicology and Cultural Heritage University of Pavia Corso Garibaldi 178 26100 Cremona Italy; ^4^ Department of Drug Sciences University of Pavia Via Taramelli 12 27100 Pavia Italy; ^5^ Centro Grandi Strumenti University of Pavia Via Bassi 21 27100 Pavia Italy; ^6^ Department of Physics University of Pavia Via Bassi 6 27100 Pavia Italy; ^7^ Department of Heritage Science and Technology Studies Graduate School of Korea Heritage Korea National University of Heritage Buyeo, Chungcheongnam-Do 33115 Korea

**Keywords:** Alginate hydrogel, Chemical crosslinking, Biopolymer, Polyaziridine, Gel application

## Abstract

Alginate biopolymer is widely employed in many industrial fields thanks to its pleasing features of biodegradability, biocompatibility, low toxicity, and relatively low cost. The gelling process of alginate with divalent cations is fairly simple and thus it is used as a versatile biomaterial to tailor the desired mechanical and moisture properties. This study focused on developing new gel formulations to enhance the properties of calcium‐alginate hydrogel (CA). The newly synthesized hydrogels, referred to as CA‐CHEM gels, were chemically cross‐linked with different ratios of pentaerythritol tris[3‐(1‐aziridinyl)propionate] (PTAP) through the reaction between the carboxylic groups of alginate and aziridines of PTAP. The reaction was successfully monitored by NMR. The new CA‐CHEM gels were chemically characterized using FTIR‐ATR, while SEM analysis confirmed the changes in the porosity and homogeneity of the network. Additionally, thermogravimetric analyses and mechanical properties showed improvement in degradation stability and in structural strength, compared to plain CA, with an increasing PTAP content up to 1 % w/w. Finally, the new CA‐CHEM gels effectively controlled water absorption and release. In particular, CA‐CHEM‐1 performed as the most controlled system, making it promising for delivering aqueous cleaning solutions on water‐sensitive surfaces such as a wooden historical musical instrument.

## Graphical abstract

A set of novel alginate‐based gel chemically cross‐linked with pentaerythritol tris[3‐(1‐aziridinyl)propionate] (PTAP) were synthesized to enhance chemical and mechanical properties. The chemical crosslinking improved degradation stability, structural strength, and control over water absorption and release, compared to the precursor ionically linked calcium‐alginate. One specific gel formulation showed promise for cleaning of water‐sensitive artwork's surfaces offering great usability and control.

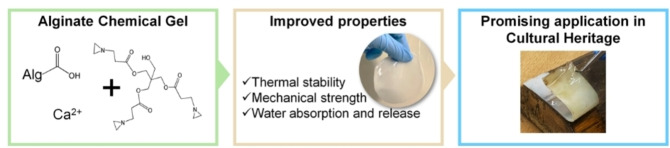

## 1 Introduction

Alginate (Alg) is a natural polysaccharide extracted from different species of brown seaweed and several bacteria. It contains blocks of β ‐D‐mannuronic acid and ‐L‐guluronic acid residues linked by 1→4 glycosidic bonds.[^1^, ^2^] Due to its biocompatibility, biodegradability and good processability with cations,^[2]^ this natural polymer has attracted increasing interest in many applications. Indeed, alginate was used as a coating for fruits and vegetables, emulsifying agent in the food industry, injectable hydrogel for tissue engineering, wound dressing system in biomedical sciences, as a drug and protein delivery system in pharmaceuticals and as an eco‐friendly surface cleaning tool in the field of cultural heritage.[^3^, ^4^, ^5^, ^6^, ^7^, ^8^]

Numerous studies highlighted the prompt gel‐forming properties of alginate with divalent cations (Ca^2+^, Sr^2+^, Ba^2+^, etc.), in particular with calcium ions, which form an “egg‐box” 3D network.[^9^, ^10^, ^11^] This process does not require harsh conditions or potentially toxic reagents; in this regard, it has the greatest application as an excipient in drug delivery, which allows gradual loading and release of the drug through the process of diffusion and erosion.[^12^, ^13^, ^14^, ^15^, ^16^] Despite these advantages, the standard mixture of alginate and calcium cations may result in poorly controlled mechanical properties, which are unsuitable for manufacturing large‐scale hydrogel due to the inhomogeneity of gelification by “egg‐box” structure, and limited long‐term stability.[^17^, ^18^] All these drawbacks potentially limit its applications particularly in the field of cultural heritage conservation.[^8^, ^19^, ^20^, ^21^]

A way to improve alginate hydrogel resistance is by covalent crosslinking either with polymers or other compounds to trigger the strength and elasticity of the network or its porosity.[^6^, ^22^, ^23^, ^24^, ^25^] For instance, one study reported that the porosity of alginate hydrogels can be controlled by the design of the cross‐linkers (two different types of cross‐linkers were used: disulfide‐tetrazine and disulfide‐maleimide).^[23]^ Moreover, this study confirmed that the cross‐linker proportion and porosity were significant factors influencing drug release behavior of the alginate hydrogels. Zhu et al. synthesized a pericellular matrix‐like material using alginate‐amino acid derivatives in combination with poly(dl‐glycolic acid) and reported that the modified alginate accelerated cell growth.^[22]^ Numerous studies investigated the bioactivity of alginate‐peptide/protein conjugates and showed their usefulness in a range of potential applications.^[22]^ All these studies reported that the chemical modifications or chemical crosslinking provided similar or improved properties to the alginate biopolymer depending on cross‐linker, amount of cross‐linker, reaction conditions, and compatibility with solvents. Hence, there are several factors to be controlled to tailor a good hydrogel, although most of the studies are limited to medicinal and pharmaceutical fields.

Therefore, this research aimed to develop new chemically cross‐linked gels which can be used as smart cleaning tools for removing undesired materials from different artefact surfaces. The newly synthesized gels were named as CA‐CHEM gels. The focus was on enhancing the mechanical and chemical properties of the resulting gel systems by reacting the carboxylic groups of alginate with the aziridine rings of pentaerythritol tris[3‐(1‐aziridinyl)propionate] (PTAP). The concept was to create new chemically cross‐linked alginate hydrogel with polyaziridines while still utilizing the traditional ionic crosslinking process with Ca^2+^ ions, in a two‐step gelation process. In general, aziridines are reactive substances in ring‐opening reactions with many nucleophilic substances, and PTAP is widely used as a cross‐linker and modifier to improve polymers’ mechanical properties, durability, and chemical stability.[^26^, ^27^, ^28^, ^29^, ^30^] For instance, PTAP can react with the alginate carboxylic functions, opening aziridine rings and forming new ester groups.

In this regard, the gel's physicochemical features were determined to verify the hypothesis. The first‐step chemical crosslinking process between alginate and polyaziridine was examined with liquid‐state Nuclear Magnetic Resonance (NMR) analysis; after a second‐step ionic crosslinking between Alg‐PTAP with Ca^2+^ ions analysis of the gels were carried out with Fourier transform infrared spectrometry with a universal attenuated total reflectance accessory (FTIR‐ATR). The gel's morphology, thermal stability, moisture and mechanical properties were also investigated to assess its potential applications.

As a promising application, we employed the most suitable newly formulated gel (among the three different chemical compositions of CA‐CHEM gels) for the cleaning of a 19^th^ century stringed musical instrument. The conservation of historical wooden musical instruments often requires cleaning and removal of surface contaminants or past restoration materials. However, these processes represent a delicate operation because they may irreversibly compromise the artefact if it is not correctly performed.[^31^, ^32^, ^33^] Therefore, the cleaning process must be safe for the artefact, ensuring a controlled action and typically, hydrogel limits water or solvent spread on the artefact's surface. Here, we used CA‐CHEM gel to selectively detach a paper label from the wood and remove the glue residue without risking damage to the label or the underlying wooden surface with excessive water diffusion.

## Materials and Methods

### Materials

Sodium alginate (Alg) medium viscosity (CAS 9005–38–3) Mw ~80,000 to 120,000 g/mol was purchased from Merck Life Science S.r.l. (Milan, Italy), and calcium chloride anhydrous prills (CaCl_2_) was supplied by Fluorochem Ltd (Hadfield, UK). Pentaerythritol tris[3‐(1‐aziridinyl)propionate] (CAS 57116–45–7, 10 % (w/v) in water), provided by ICHEMCO srl (Milan, Italy), was used as a crosslinking agent. Whatman^®^ qualitative filter paper, Grade 1 (WHA1001090) was used for the moisture property examinations. Water was purified using a Millipore Organex system: R ≥18 M cm (Burlington, MA, USA).

### Gel Preparation

Aqueous sodium alginate solution 2 % (w/v) was prepared as previously reported.^[34]^ In brief, Alg (0.2 g) was completely dissolved in 10.0 mL of distilled water in a stoppered glass flask at 40 °C. Three Alg solutions were prepared as mentioned above and a different amount of PTAP (1, 2, and 4 mg, corresponding to 0.5, 1.0, and 2.0 % w/w respect to the polymer) was added to each alginate solution. Considering the formal molar weight of sodium alginate monomer (198.11) and the molar weight of the tris‐aziridine (427.49), the molar ratio between alginate monomer (or carboxylic group) and PTAP was approximately 50 : 1, 25 : 1, and 12.5 : 1, respectively. The reaction mixtures were magnetically stirred at 50±2 °C for 30 minutes. To prepare thin gel films, each Alg‐PTAP solution was poured into Petri dishes, maintaining the thickness around 2–3 mm. Subsequently, 2 % (w/v) CaCl_2_ aqueous solution (about 20 mL) was slowly added into the Petri dishes, and the interaction between polymer and calcium ions was allowed for 15 minutes. After formation of gels, they were washed several times in deionized water to remove unreacted materials and named as CA‐CHEM‐0.5, CA‐CHEM‐1, and CA‐CHEM‐2 according to the different amounts of PTAP used for crosslinking. For comparison, CA gel (Alg solution crosslinked with CaCl_2_) was prepared using the same procedure but without PTAP in the formulation.

### Physicochemical Characterization of Gels

#### NMR Investigation of the Crosslinking Process

Liquid‐state Nuclear Magnetic Resonance (NMR) analyses were conducted on Alg‐PTAP materials to assess the aziridine ring opening of PTAP and its subsequent reaction with Alg. The sample was prepared with 0.5 % of PTAP (molar ratio Alg monomer:PTAP=50 : 1) in D_2_O and the resulting turbid/colloidal solution was further diluted 1 : 5 in D_2_O. The analysis was performed by a Bruker AVANCE 700 MHz spectrometer equipped with a TCI cryoprobe. The acquisition of spectra was carried out at 298 K, with the chemical shift standard to TSP (3‐(Trimethylsilyl)propionic‐2,2,3,3 acid sodium salt).

#### Gel Characterization

The solid gel films, obtained after partial chemical crosslinking of Alg with PTAP and further ionic crosslinking with CaCl_2_, were analysed by different techniques. Infrared spectra were measured by the Perkin‐Elmer Spectrum 100 FT‐IR spectrometer equipped with an ATR accessory (PerkinElmer, Waltham, MA, USA) with diamond crystal. IR spectra were collected in the range between 550 and 4000 cm^−1^ with a resolution of 4 cm^−1^, subsequently expressed in absorbance units. Prior to FTIR analysis, gel samples were fully dried inside a vacuum desiccator (with a slow vacuum flow) for approximately 5 hours. The morphological characteristics of CA and CA‐CHEM gels were observed by a scanning electron microscope (SEM) Tescan MIRA 3XMU series field emission FE‐SEM microscope (TESCAN, Brno, Czech Republic), operating at an accelerating voltage of 5 kV in high vacuum, and located at the Arvedi Laboratory, CISRiC‐University of Pavia, Italy. To analyse the gel samples by SEM, they were fully dried inside a vacuum desiccator (with a slow vacuum flow) for approximately 5 hours, and then, they were graphite‐sputtered for 20 seconds using the Cressington Carbon Coater 208 C.

#### Thermal Stability Measurements

The thermogravimetric analysis (TGA) was performed with Q5000 equipment (TA Instruments, New Castle, DE, USA) interfaced with a TA5000 data station under nitrogen flow (10 mL min^−1^) by heating approximately 3 mg of samples (dry solid films) in a platinum plate from room temperature to 600 °C at a heating rate of 10 °C min^−1^. The gravimetric mass changes and corresponding temperature values for each phase were defined by considering the derivative thermogravimetric (DTG) curve calculated by TA Instruments’ data analysis software.

#### Mechanical Properties

CA and CA‐CHEM gels (hydrated gel films) were stretched uniaxially to evaluate their tensile behaviour with a TA.XT plus analyser from Stable Micro Systems Godalming, UK. The analyser was equipped with a 5 kg load cell. Before starting with the analysis, the film thickness was measured using the Sicutool 3955G‐50 (Sicutool, Milan, Italy). Afterwards, each thin gel film (1 cm ×3 cm) was clamped on an A/TG tensile probe with the initial distance between the clamps set at 1 cm. Subsequently, the top grip was raised at a constant speed of 2 mm/min to a distance of 50 mm. After testing, the tensile strength and elongation at break were calculated; three measurements were carried out for each gel. The same analysis was performed on aged gel, after storing for 60 days at 4 °C, to evaluate the shelf‐life of the CA‐CHEM gels.[^5^, ^18^]

#### Moisture Properties and Gel Fraction

Gel content/fraction (GF) was calculated according to the following Equation 1:[^31^, ^33^]
(1)

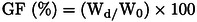




Where W_d_ is the dry weight of the gel (final product) and W_0_ is the weight of the materials in the initial reaction mixture (reactants).

Water absorption and releasing capacities were measured by calculating the Swelling Capacity (SC), the Water Release (WR), and the correlation between water release and water absorption (WR/WA) by using Equations (2), (3), and 4.[^6^, ^33^]
(2)

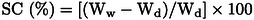




W_d_ is the dry weight of the gel, and W_w_ is the wet weight of the gel, which is a freshly prepared gel when fully loaded with water.
(3)

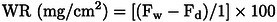




F_d_ is the initial weight of the dry filter paper, and F_w_ is the weight of the wet filter paper after 30 min of contact with the gel.
(4)

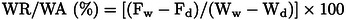




As the gel swells due to the water absorption, the value of swelling capacity (SC%) is related to the water absorption (WA).

### Gel Application to a Real Case: Leopold Noiriel's Historical Double Bass

**Figure 1:**
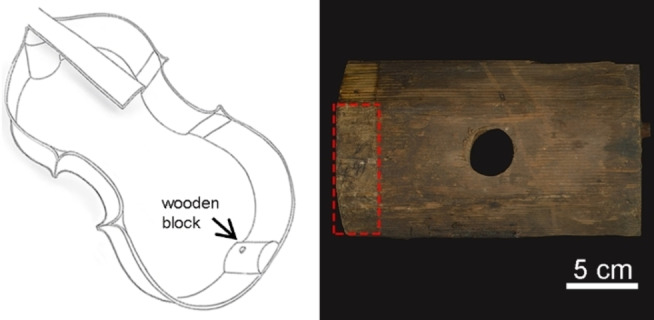
A sketch of the internal side of the double bass (on the left) and the wood bottom block of the instrument (on the right) with the label highlighted by a red rectangle.

To explore the potentiality of a newly formulated chemical gel in the confined and controlled loading of water‐solvents over water‐sensitive surfaces, CA‐CHEM‐1 was selected and tested on the historical wooden double bass of Leopold Noiriel (1789–1849), which was made in 1830 (Figure 1). The gel was loaded with water for 24 h and then applied to carefully remove a paper label glued to the bottom wood of the double bass. For the first application, the gel was applied for 30 minutes to gently remove the label with a tweezer and spatula. For the second application, the gel was applied for another 30 minutes, after, a scalpel and swab were used to remove the softened residues of animal glue (Figure S1).

After the gel application, the efficacy of the removal of both the paper label and glue residues was assessed by a non‐invasive multispectral imaging (MSI) system developed by Profilocolore srl (Rome, Italy).^[35]^ Images were captured using a Nikon Z8 45.7‐megapixel mirrorless digital camera (Minato, Tokyo, Japan) with an AF−S 50 mm 1 : 1.4 G Nikkor objective (Minato, Tokyo, Japan). UVIF images (f/8 as focal ratio, ISO 400, 25 s exposure time) were acquired by exposing the UV LED lamps (365 nm) and using the PFCL UV‐IR cut (400–700 nm) optical filter with the PFCL−A filter. During the acquisition and image processing, the software packages Capture Pick®, Spectra Pick®, and Pick Viewer® were used. Furthermore, Pick Viewer's likelihood tool was used to display all the pixel with spectral similarities in a gray‐scale image, where the value 1 corresponds to the maximum similarity.

In addition to MSI, External Reflection Fourier‐Transform Infrared spectroscopy (ER‐FTIR)^[36]^ was carried out with an Alpha‐R Bruker portable instrument (Bruker Optics, Billerica, MA, USA) to monitor the removal of the glue and exclude the gel residues after cleaning. Spectra were collected between 7500 and 375 cm^−1^ at a resolution of 4 cm^−1^ and acquisition time of 1 min scans. Spectra acquisition and their transformation from reflectance to absorbance by the Kramers–Kronig (KK) algorithm was processed by OPUS 7.2 software ver. 7.2.

## 2 Results and Discussion

### 2.1 Chemical Modifications Due to the Crosslinking

The gelation process involves two different subsequent steps: (i) a reaction between Alg with PTAP, during which the aziridine groups of PTAP undergo ring‐opening, forming new bonds with carboxylate groups of Alg and resulting in the formation of new ester functions. Due to the limited amount of PTAP, this reaction induces only a partial crosslinking, leaving most carboxyl groups of Alg still available; (ii) ionic crosslinking with CaCl_2_ induced by electrostatic interactions between calcium ions and residual carboxylate groups.

The chemical modification of Alg was investigated after the first step of the gelation process to assess that the reaction between aziridine and carboxylate groups had occurred.

**Figure 2:**
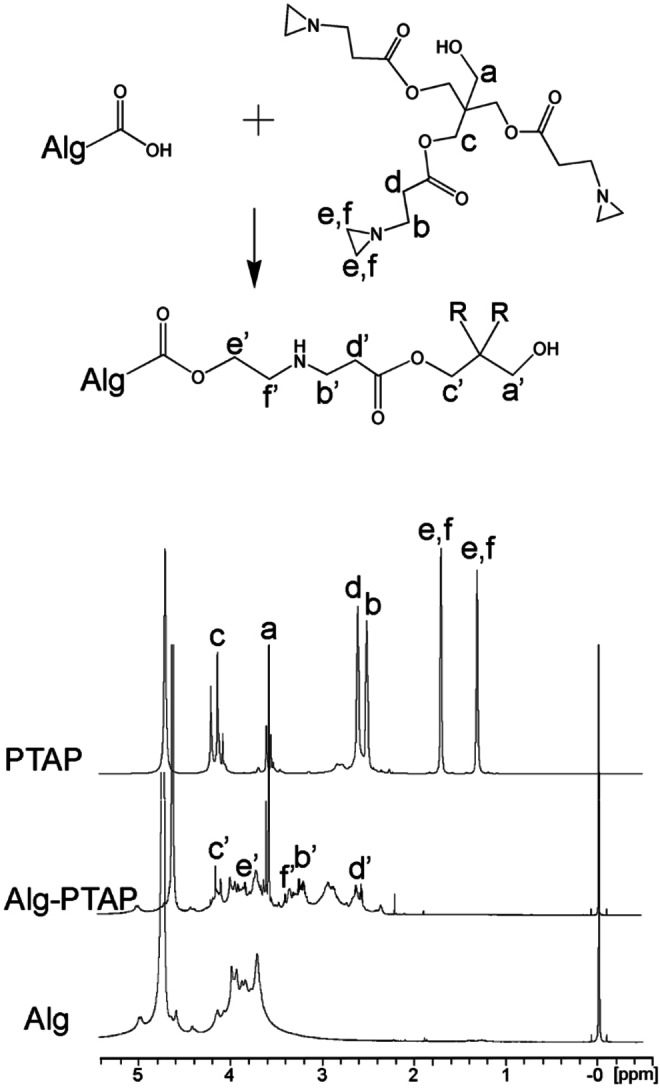
^1^H NMR spectrum of Alg (sodium alginate), PTAP (pentaerythritol tris[3‐(1‐aziridinyl)propionate]), and Alg‐PTAP. Characteristic peaks are denoted.

Liquid state NMR analysis performed on the turbid/colloidal solution of the PTAP‐modified alginate polymer^[37]^ confirmed the formation of new ester groups in CA‐CHEM‐0.5 resulting from the reaction between Alg and polyaziridine. Results are resumed in Figure 2, where the assignment of the ^1^H‐NMR peaks is shown. Peaks e, f, at δ 1H 1.73 ppm and δ at 1H 1.33 ppm, corresponding to the hydrogen atoms of the aziridine ring, disappeared after the reaction, which indicated the aziridine ring opening to form a secondary amine.[^38^, ^39^, ^40^] The assignment of e’, f’, b’, and d’ peaks was confirmed by comparison of 2D COSY and HSQC spectra of the three compounds considered (Alg, PTAP and Alg‐PTAP). The COSY correlation peaks between e’, f’ and between b’, d’ (Figure S2) demonstrated covalent bonding, indicating successful crosslinking.

### 2.2 FTIR Study on Gel Materials

**Figure 3:**
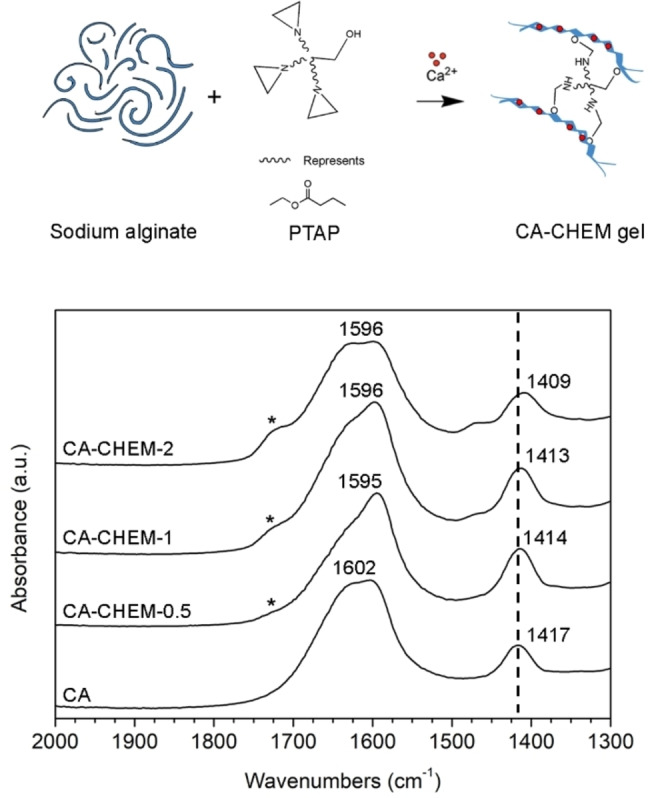
Graphical representation of the chemical structure of CA‐CHEM gels and reagents (top) and FTIR‐ATR spectra of the gels prepared with different PTAP content, in the range 2000–1300 cm^−1^. Ester bands are marked with an asterisk at 1725 cm^−1^.

FTIR analysis was done on solid dry gel films to further investigate the chemical modification of original gel material (CA) due to the crosslinking processes in Figure 3. In accordance with the FTIR of alginate polymer, all the spectra of CA‐CHEM gels (0.5, 1, and 2) showed broad peaks from 3600 cm^−1^ to 3000 cm^−1^ related to the O−H bonds, around 1600 cm^−1^ and 1410 cm^−1^ attributed to the symmetric and asymmetric stretching vibrations of COO^−^, respectively, and at 1030 cm^−1^ for C−O bonds.[^11^, ^17^, ^41^] Full‐range spectra and band‐assignment are available in Figure S3 and Table S1. Compared to CA, a band at approximately 1725 cm^−1^ (corresponding to ester C=O) appears in the spectra of the chemically modified gels, with its intensity increasing as the amount of PTAP increases. The new band, which is more clearly observable in the spectra of CA‐CHEM‐1 and CA‐CHEM‐2 in Figure 3, can be related to the formation of new ester groups.[^26^, ^29^, ^38^, ^39^] It should be considered that plain PTAP itself contains ester groups, therefore, the appearance of new bands in the IR spectra of CA‐CHEM materials cannot be considered as unique evidence of the reaction involving carboxylate and aziridine groups. However, the results of liquid state NMR experiments provided clear evidence of PTAP aziridine rings opening and the formation of new ester groups on the Alg polymer chains (see section 2.1). Moreover, the symmetric stretching vibrations of COO^−^ observed in the CA‐CHEM materials shifted to lower wavenumbers (from 1414 to 1409 cm^−1^) as the amount of PTAP increased. This result could be attributed to the interaction of carboxylate groups of Alg and calcium ions: as the PTAP amount and the chemical crosslinking increase, the carboxylate groups available for ionic interactions and their degree of interaction with Ca^2+^ decrease.[^17^, ^42^]

### 2.3 Effect of Chemical Crosslinking on Morphology and Thermal Stability

**Figure 4:**
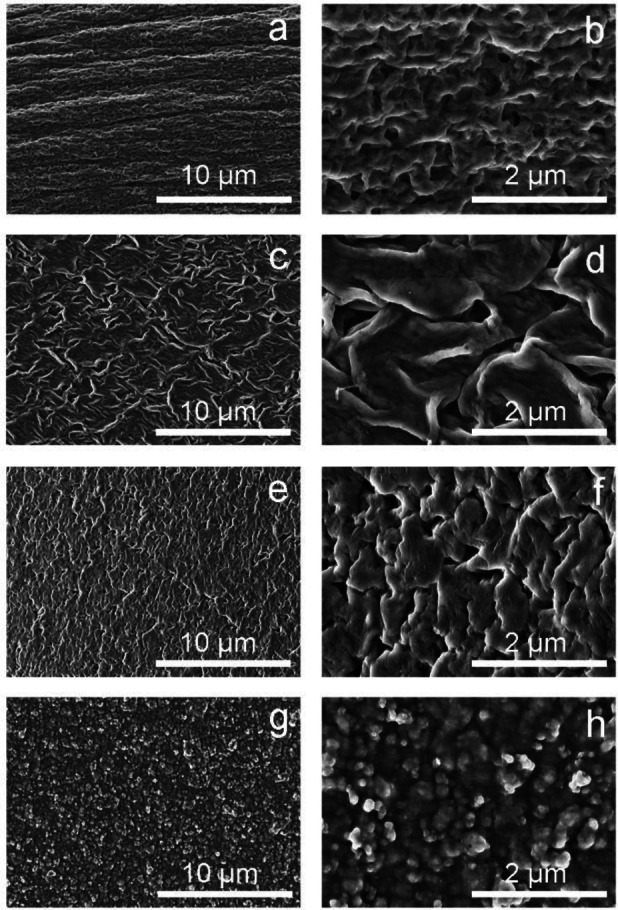
SEM images of the surface of CA (a‐b), CA‐CHEM‐0.5 (c‐d), CA‐CHEM‐1 (e‐f), and CA‐CHEM‐2 (g‐h) at different magnifications.

The morphological and micro‐structural characteristics of the new CA‐CHEM gels, compared to the plain CA gel, were examined by SEM experiments (Figure 4). CA gel showed an irregular surface probably due to the formation of an “egg‐box” structure.[^17^, ^41^, ^42^, ^43^] Materials obtained after functionalization with PTAP showed a more regular and compacted appearance. These morphological features were mostly visible in CA‐CHEM‐0.5 and CA‐CHEM‐1, where the gel surfaces appeared quite homogeneous, suggesting that the reaction between the cross‐linker and the original biopolymer (Alg) occurred extensively. On the contrary, CA‐CHEM‐2 showed different morphological features with aggregated particles all over the surface, which may be attributed to the higher PTAP content in the Alg reaction mixture and to the higher level of crosslinking in the polymer matrix, or to the polymerization‐induced phase separation of the PTAP with carboxylate groups of Alg.[^30^, ^44^] The SEM results clearly showed that the high concentration of the crosslinking material (2 % w/w, corresponding to a molar ratio sodium alginate monomer: PTAP=12.5 : 1) was unsuitable for the preparation of a good‐performance material. Finally, the varying surface structures of CA‐CHEM gels may be explained on the basis of the different degrees of interaction with calcium cations and the consequent different level of chemical crosslinking, as also supported by the FTIR results discussed in section 2.2.

**Figure 5:**
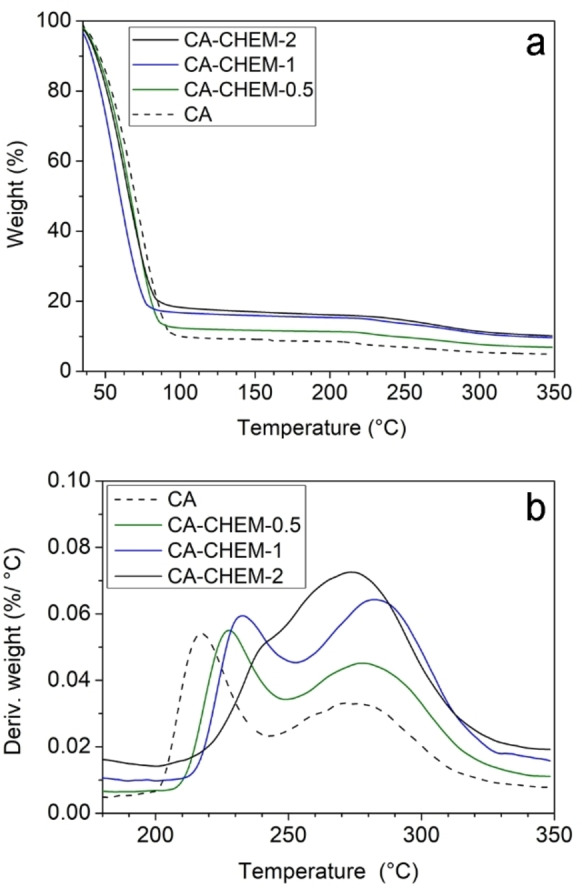
TGA curves from room temperature to 350 °C (a) and DTG traces from 180 °C to 350 °C (b) curves for CA and CA‐CHEM gels obtained with different PTAP content.

Thermogravimetric profiles and related DTG curves (Figure 5) indicated a three‐stage decomposition occurred in all the analyzed gels. The first mass loss between 50 °C and 100 °C, is the most intense (up to 90 %), and it is due to the moisture evaporation.[^7^, ^45^] When the ratio of PTAP increased in the formulation of the CA‐CHEM gels, the weight loss for this stage considerably decreased (from 90 % to 82 %), probably due to lower water transferring attitude introduced by the crosslinking^[46]^ (Table 1). The second stage occurred between 200 °C and 250 °C, attributed to tightly bound water,[^47^, ^48^] while the third stage, ending at 350 °C, could be associated with the decomposition of alginate.[^47^, ^49^] For both the second and third stages, as better evident from the DTG signals (Figure 5b), the increase of PTAP ratio in the formulation of CA‐CHEM gels delayed the degradation at higher temperatures, pointing out the thermal stability improvement.[^25^, ^49^, ^50^] The delayed degradation could be attributed to the chemical crosslinking occurred between polyaziridine and alginate biopolymer, as confirmed by NMR (in section 2.1). From the TGA curves and the related values reported in Table 1, it is possible to conclude that by increasing the PTAP content in the samples: ‐ the amount of adsorbed water sensibly decreases (from 91 % for the pure CA to 82 % for CA‐CHEM‐2) and the maximum temperature for the dehydration process decrease; ‐ the amount of chemically bonded water increases, as its release temperature (the maximum of the process rate shifts from 205 °C for pure CA to 228 °C for CA‐CHEM‐2); ‐ the mass loss decreases and hence the amount of residual sample after the heating treatment increases: this could be due to the formation of a stable PTAP‐Alg‐Ca^2+^ complex in increasing amount by increasing the linking agent. Globally, as evident from Figure 5, CA‐CHEM‐1 results more thermally stable than the other samples, having the maximum temperature for the third step and the residual final mass higher than the values for all the other examined materials (the residual mass value coincides, within the technique experimental error, with the one of the CA‐CHEM‐2 gel).

**Table 1:** Thermogravimetric analysis (TGA) data obtained for CA and CA‐CHEM gels.

Gels	1^st^ Step		2^nd^ Step		3^rd^ Step		% Residue at 350 °C
	Δ Mass loss (%)	T max (°C)	Δ Mass loss (%)	T max (°C)	Δ Mass loss (%)	T max (°C)	
CA	90.8	92	0.6	205	1.4	273	2.2
CA‐CHEM‐0.5	88.2	90	0.6	216	1.6	278	2.9
CA‐CHEM‐1	83.7	84	1.2	222	1.8	281	3.6
CA‐CHEM‐2	82.1	77	2.2	228	1.9	275	3.7

### 2.4 Mechanical Improvements

**Figure 6:**
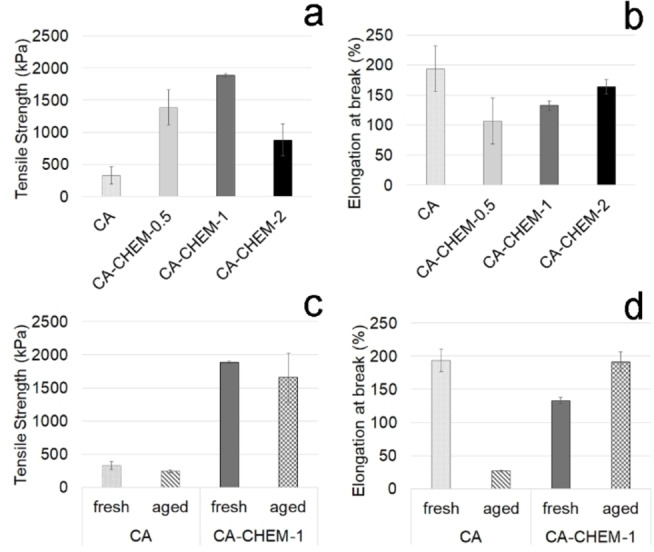
Mechanical properties determined by tensile test (mean value±s.d.; n=5) of gels CA and CA‐CHEM‐0.5, 1, and 2 (a‐b), and fresh and aged CA and CA‐CHEM‐1 (c‐d).

The results of the tensile test (Figure 6) evidenced that the chemical crosslinking improved the mechanical properties of the new CA‐CHEM gels when compared to plain CA. It is well‐known in the literature[^23^, ^38^, ^51^] that covalent cross‐links remain stable under deformation, resulting in larger stress resistance than physical ones. In the present work, the addition of PTAP substantially and markedly increased the hardness of CA‐CHEM gels. More precisely, a significant increase in tensile strength was observed for CA‐CHEM‐0.5 and CA‐CHEM‐1 with respect to plain CA. In the case of CA‐CHEM‐2, although larger than CA, it was considerably lower than those of the other chemical gels (Figure 6a). This suggested that the addition of increasing PTAP concentration above a critical value (>1 % w/w, corresponding to a molar ratio sodium alginate monomer: PTAP=25 : 1) was responsible for a significant decrease of gel tensile strength. The aforementioned results may be explained by an excessive amount of the cross‐linker that could limit polymer chain flexibility and weaken the gel structure.

As seen in Figure 6b, the chemical crosslinking between CA and PTAP produced a reduction of the elongation of the gels compared to plain CA. However, all the modified gels showed sufficient resistance to the applied force.

It is important to emphasize that, for cleaning applications, increasing tensile strength while reducing flexibility enhances the gel network's stability and resistance to deformation, significantly minimizing the risk of leaving gel residues on the surface. From this perspective, since CA‐CHEM‐1 demonstrated the best mechanical properties, additional analyses were performed after 60 days of ageing (Figure 6c,d). Results confirmed that CA‐CHEM‐1 preserved a high tensile strength even after two months from its preparation, while CA gel showed a rapid de‐structuring behavior due to the aging process.

These results indicate that crosslinking with PTAP considerably improved the mechanical properties of alginate‐based gels and made them to be substantially kept at least up to 60 days of shelf‐life.

### 2.5 Moisture Properties and Gel Fraction of the Gels

The data concerning the moisture properties of CA and CA‐CHEM gels are summarized in Table 2. The swelling capacity values (SC‐%) indicated that all CA‐CHEM gels absorbed less water amount than CA gel, as pointed out also by the thermogravimetric data in section 2.3. In particular, the lowest absorption was observed for CA‐CHEM‐1, and the decrease was around 53 % compared to plain CA. The highest water absorption and the lowest water release (WR) displayed by CA gel could be explained by its crosslinking process with Ca^2+^ which provided hydrophilic behavior to the gel matrix, where Ca^2+^ ions and carboxylate groups can interact with the water molecules in the gel system. For CA‐CHEM gels, moisture properties seemed strictly dependent on the PTAP concentration, and consequently, on the extent of the resulting crosslinking process.

**Table 2:** Moisture properties of CA and CA‐CHEM gels: swelling capacity (SC), water release (WR), and ratio between water release and water absorption (WR/WA); (mean value±s.d.; n=5).

Gel	SC (%)	WR (mg/cm^2^)	WR/WA (%)
CA	29.3 (±1.2) ×10^2^	8.4 (±1.0)	16.7 (±1.6)
CA‐CHEM‐0.5	19.3 (±2.1) ×10^2^	18.8 (±2.2)	29.1 (±6.9)
CA‐CHEM‐1	13.6 (±2.5) ×10^2^	15.1 (±2.4)	16.2 (±1.6)
CA‐CHEM‐2	15.8 (±0.8) ×10^2^	28.3 (±8.9)	42.2 (±5.6)

For instance, CA‐CHEM‐0.5 contains the lowest concentration of PTAP (0.5 % w/w), and the number of aziridine groups may not be enough to obtain optimal crosslinking with carboxyl groups of Alg. The unreacted carboxyl groups could be cross‐linked with Ca^2+^ ions, keeping the ability to retain water. On the other hand, CA‐CHEM‐2 (PTAP 2 % w/w) resulted in quite rigid gel, which showed the highest water release. This behavior may be ascribed to the larger amount of carboxylic groups of Alg reacted with aziridines of PTAP and to the consequent lower number of ionic species (carboxylate and Ca^2+^ ions) available to retain water into the matrix. Consequently, the gel is more prone to release water when applied to a surface. The enhancement of covalent crosslinking, and the consequent decrease of ionic sites in CA‐CHEM‐2 is likely responsible also for the compact morphology with aggregates observed by SEM, as discussed in section 2.3.

In the case of CA‐CHEM‐1 (PTAP 1 % w/w), the lowest water release among the new gels was observed. It may be attributed to the most favorable concentration of PTAP to achieve a stable crosslinking between Alg and PTAP,^[26]^ which decreases the water mobility, as well as to the regular surface and well‐defined porosity of the gel described in section 2.3. In accordance with this hypothesis, it is worth noting that CA‐CHEM‐1 also showed the highest tensile strength (1886±33 kPa) among the set of gels, which is more than 5 times higher than CA, implying that it is more resistant due to the appropriate crosslinking process (Figure 6).

Moreover, concerning the WR/WA results, crosslinking with PTAP influences the capability of controlling water release. CA‐CHEM‐1 is a promising gel material displaying an effective control similar to CA, which results in reduced water uptake and minimal release. This is a vital consideration when applying gel materials on the water‐sensitive surface of artefacts.

The gel fraction (GF) values were also determined for all the gel materials based on PTAP‐modified Alg as well as for plain CA for comparison. In all cases similar GF values (>95 %) were observed.

### 2.6 Water Delivery Behavior and Cleaning Efficacy

CA‐CHEM‐1 proved to have good mechanical properties that were maintained over a shelf‐life of 60 days. Furthermore, the chemical crosslinking with PTAP influenced the polymer's hydrophilic properties providing desirable control of water absorption/release. For these reasons, the gel was preliminarily applied to control the delivery of water over the surfaces of water‐sensitive artefacts (Figure S1). Historical wooden musical instrument known as Leopold Noiriel's double bass was chosen for the test.

**Figure 7:**
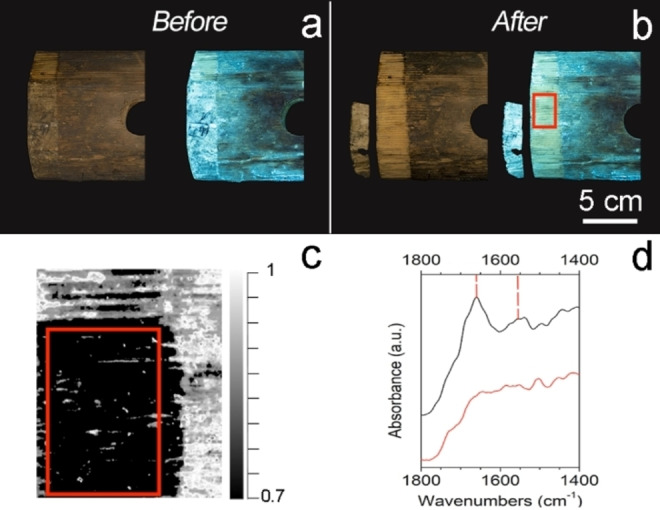
Images before (a) and after (b) cleaning the Leopold Noiriel's double bass wood block in visible and UV light, and after processing the image with the likelihood tool (c), with the gel application area in red square; (d) ER‐FTIR spectra after Kramers‐Kronig transform of uncleaned (black) and cleaned (red) area. The marker bands of amide I and II of the animal glue are represented with dotted red lines.

At first, CA‐CHEM‐1 loaded solely with water was applied to detach a paper label firmly glued to the wood surface; in the second application, the gel was applied to selectively remove the glue residues from the same wooden surface (Figure 7). After the first application, the paper label was successfully removed without using harsh mechanical action (Figure S1). After the second application, the animal glue residues on the wood were considerably reduced by the selective and controlled gel cleaning. The UV image processed by the maximum‐likelihood tool in Figure 7c, gives a clear distinction between unclean and cleaned areas (corresponding to 1 and 0.7 values in the grayscale, respectively), where some glue residues are still present. In addition, ER‐FTIR measurements were performed on the treated area to monitor the removal of the glue from the wood surface. ER‐FTIR spectra (Figure 7d) clearly showed the intensity decrease (if not disappearance) of the bands at 1660 and 1550 cm^−1^, (corresponding respectively to amide I and amide II absorptions, in protein derivatives^[6]^) after the cleaning procedure. It confirmed that the application of gel induced the almost exhaustive removal of old animal glue from the surface of the considered wood instrument.

## 3 Conclusions

In this study, we developed novel alginate hydrogels, named CA‐CHEM gels, by chemically crosslinking carboxylic groups of alginates with aziridine groups of pentaerythritol tris[3‐(1‐aziridinyl)propionate] (PTAP). Then, ionic crosslinking involving unreacted carboxylic groups and Ca^2+^ ions was also carried out to obtain the final material. The chemical reaction induced the aziridine rings opening and the formation of new covalent bonds with carboxylic groups to form new ester derivatives. We successfully synthesized alginate modified with various amounts of PTAP (0.5, 1, and 2 % w/w). Morphological features as well as mechanical and moisture properties of the new gels significantly differ from those of plain CA. Increase of PTAP concentration induced improvement of the thermal stability and decrease of hydrophilicity when compared to plain CA gel. Improvement of mechanical stress resistance strongly depends on the amount of cross‐linker: for instance, CA‐CHEM‐1 showed the highest tensile strength (1886±33 kPa), which is more than 5 times compared to plain CA, suggesting that the reaction involving 1 % PTAP provides the optimal level of crosslinking. Gel properties were maintained even after ageing, indicating its potential use after at least 60 days. CA‐CHEM‐1 was tested to deliver water solvents and to clean water‐sensitive surfaces, such as historical wooden musical instruments. Its flexible and resistant texture, improved moisture properties, and controlled water release (measured by the water release/absorption ratio, WR/WA 16.2±1.6 %) enabled uniform contact with the surface and efficient cleaning without affecting the underlying wooden surface. All these results suggest that, among the investigated gels, CA‐CHEM‐1 represents the most appropriate for the cleaning of water‐sensitive cultural heritage items as well as for potential application in engineering, industry, and other fields.

Moving forward, we aim to develop materials with improved biocompatibility of the chemically networked hydrogel.

## Conflict of Interests

The authors declare no conflict of interest.

4

## Supporting information

As a service to our authors and readers, this journal provides supporting information supplied by the authors. Such materials are peer reviewed and may be re‐organized for online delivery, but are not copy‐edited or typeset. Technical support issues arising from supporting information (other than missing files) should be addressed to the authors.

Supporting Information

## Data Availability

The data that support the findings of this study are available from the corresponding author upon reasonable request.
