# Supplementary material for: Chemically Crosslinked Alginate Hydrogel with Polyaziridine: Effects on Physicochemical Properties and Promising Applications
Source: Chempluschem. 2024 Dec 18;90(3):e202400649. doi: 10.1002/cplu.202400649 (PMC11912103; doi:10.1002/cplu.202400649)
Supplement: Supplementary file 1 — Supporting Information [file CPLU-90-e202400649-s001.pdf]

ChemPlusChem

Supporting Information

Chemically Crosslinked Alginate Hydrogel with Polyaziridine: Effects on Physicochemical Properties and Promising Applications

Chaehoon Lee, Giacomo Fiocco, Barbara Vigani, Teresa Recca, Chiara Milanese, Chiara Delledonne, Maurizio Licchelli, Silvia Rossi, Yongjae Chung, Francesca Volpi,* Maduka L. Weththimuni,* and Marco Malagodi

SUPPORTING INFORMATION

Chemically Crosslinked Alginate Hydrogel with Polyaziridine: Effects on Physicochemical Properties and Promising Applications

Chaehoon Lee[a,b], Giacomo Fiocco[b,c], Barbara Vigani[d], Teresa Recca[a, e], Chiara Milanese[a], Chiara Delledonne[b,f], Maurizio Licchelli[a], Silvia Rossi[d], Yongjae Chung[g] ,Francesca Volpi*[b,c], Maduka L. Weththimuni*[a], Marco Malagodi[b,c]

[a] Department of Chemistry
University of Pavia
Via Taramelli 12, 27100 Pavia, Italy
E-mail corresponding author 1*: madukalankani.weththimuni@unipv.it

[b] Arvedi Laboratory
University of Pavia
via Bell'Aspa 3, 26100 Cremona, Italy

[c] Department of Musicology and Cultural Heritage
University of Pavia
Corso Garibaldi 178, 26100 Cremona, Italy
E-mail corresponding author 2*: francesca.volpi@unipv.it

[d] Department of Drug Sciences
University of Pavia
Via Taramelli 12, 27100 Pavia, Italy

[e] Centro Grandi Strumenti
University of Pavia
Via Bassi 21, 27100 Pavia, Italy

[f] Department of Physics
University of Pavia
Via Bassi 6, 27100 Pavia, Italy

[g] Department of Heritage Science and Technology Studies, Graduate School of Korea Heritage
Korea National University of Heritage
Buyeo, Chungcheongnam-Do 33115, Korea

Corresponding authors: francesca.volpi@unipv.it; madukalankani.weththimuni@unipv.it

Summary

Figure S1: Images during the gel cleaning of the Leopold Noiriel's double bass wooden block.

Figure S2: 2D-COSY NMR spectrum of Alg (sodium alginate) functionalized with PTAP (Pentaerythritol tris[3-(1-aziridinyl)propionate]).

Figure S3: ATR-FTIR spectra of the CA-CHEMgels, prepared in different concentrations of PTAP and calcium.

Table S1: FTIR band assignments

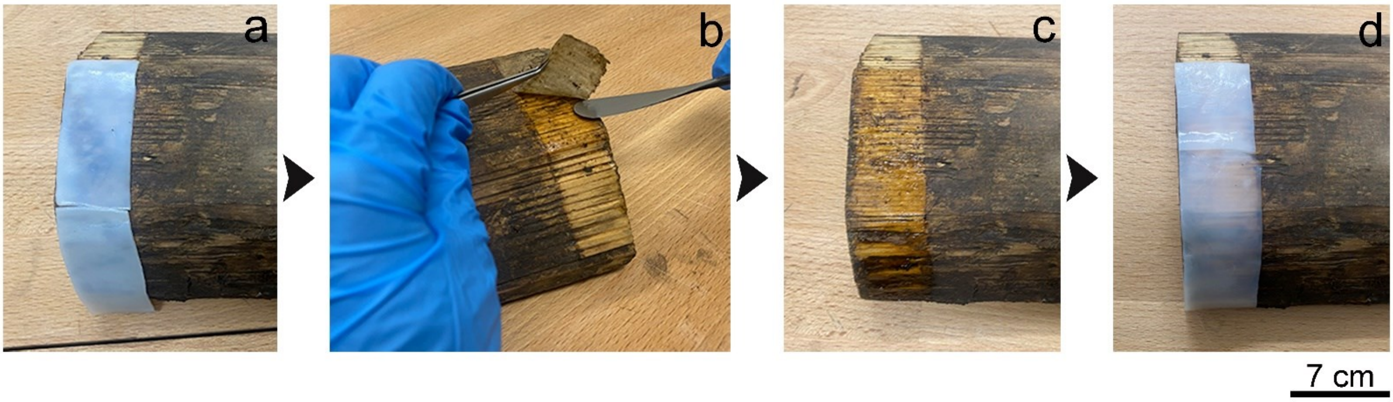

Figure S1: Images during the gel cleaning application of Leopold Noiriel's double bass wood block: first gel application for 30 min (a), gentle detachment of the paper label (b), right after removing the paper label (c), and second gel application for 30 min (d).

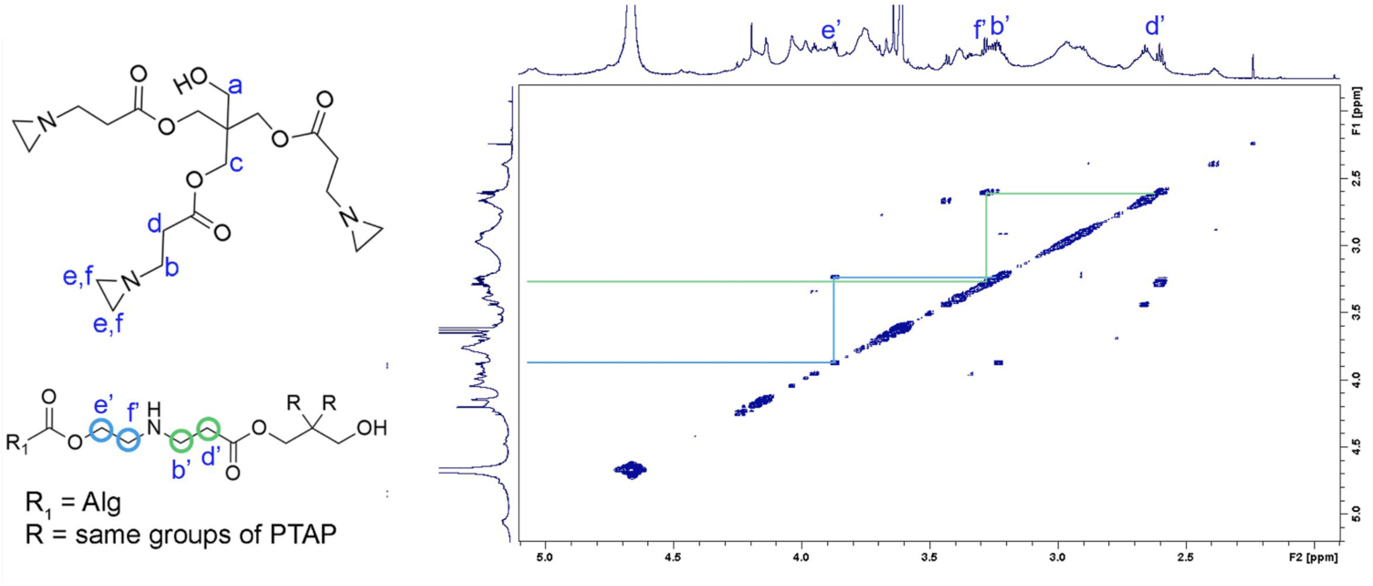

Figure S2: 2D-COSY NMR spectrum of Alg (sodium alginate) functionalized with PTAP (Pentaerythritol tris[3-(1-aziridinyl)propionate]). Blue and green lines respectively point out the connections between protons e', f' and b', d'.

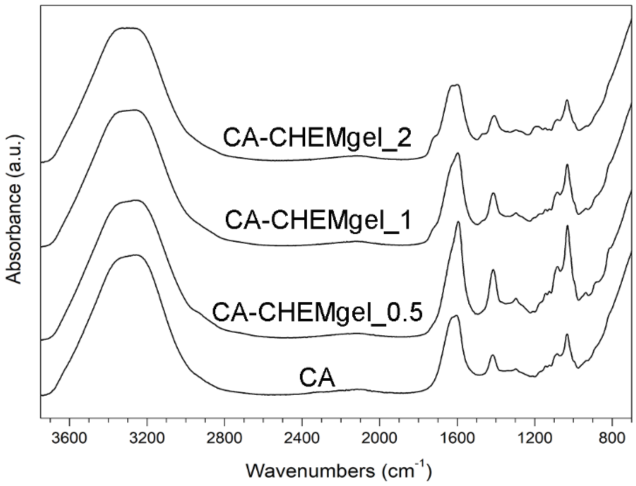

Figure S3: ATR-FTIR spectra of the CA-CHEMgels, prepared in different concentrations of PTAP and calcium.

Table S1 FTIR band assignments

**Assignment**	**Wavenumber (cm^{-1})**
vO-H	3350
$v_{as}COO^{-}$	1595
$v_{s}COO^{-}$	1409-1417
vC-O	1300, 1085, 1035
